# Quality of life before and after sympathectomy in primary hyperhidrosis: a recall-based paired analysis of 94 patients

**DOI:** 10.3389/fsurg.2026.1996976

**Published:** 2026-10-15

**Authors:** Yazeed A. Alrodiman, Qais A. Almuhaideb, Nasser M. AbuDujain, Khalid M. Alghamdi

**Affiliations:** 1 College of Medicine, Alfaisal University, Riyadh, Saudi Arabia; 2 Department of Dermatology, King Faisal Specialist Hospital and Research Centre, Riyadh, Saudi Arabia; 3 Department of Family and Community Medicine, College of Medicine, King Saud University, Riyadh, Saudi Arabia; 4 Department of Dermatology, College of Medicine, King Saud University, Riyadh, Saudi Arabia; 5 Vitiligo Research Chair, College of Medicine, King Saud University, Riyadh, Saudi Arabia

**Keywords:** Hyperhidrosis Disease Severity Scale, Hyperhidrosis Quality of Life Index, primary hyperhidrosis, quality of life, sympathectomy

## Abstract

**Objectives:**

Primary hyperhidrosis substantially impairs quality of life. Sympathectomy is an established treatment for severe focal hyperhidrosis; however, patient-reported outcomes in Arab populations remain limited. This study evaluated recalled preoperative and current postoperative quality of life after sympathectomy using validated Arabic instruments.

**Methods:**

We conducted a secondary, recall-based paired analysis of a cross-sectional survey of 94 participants with primary hyperhidrosis who reported having undergone bilateral sympathectomy. Participants completed an Arabic electronic survey between May 2024 and April 2025. Current postoperative quality-of-life scores were compared with retrospectively recalled preoperative scores using the Arabic Hyperhidrosis Quality of Life Index (HidroQoL©); severity was assessed using the Arabic Hyperhidrosis Disease Severity Scale. Wilcoxon signed-rank tests were primary; paired t tests were sensitivity analyses.

**Results:**

The mean participant age was 30.7 ± 8.9 years, and 63.8% were men. At the recalled preoperative assessment, 55 participants (58.5%) reported Grade 4 disease severity. All 94 participants provided complete paired data. The mean daily-life score decreased from 8.5 ± 2.8 to 4.9 ± 4.5 [mean paired change −3.6; 95% confidence interval (CI) −4.5 to −2.8; *d_z_* = −0.88]. The mean psychosocial score decreased from 16.9 ± 5.5 to 8.8 ± 8.7 (−8.1; 95% CI −9.7 to −6.4; *d_z_* = −0.97). The total score decreased from 25.4 ± 7.5 to 13.7 ± 12.8 (−11.7; 95% CI −14.1 to −9.3; *d_z_* = −0.98); all *p* < 0.001. Thirteen participants (13.8%) reported a higher total score after surgery.

**Conclusions:**

Participants reported substantially lower hyperhidrosis-related daily-life and psychosocial impairment after sympathectomy. Recall bias may affect the magnitude and direction of these differences, which do not establish causal treatment effects. Prospective studies incorporating true preoperative baseline assessment, operative details, compensatory sweating, and longer follow-up are needed to confirm the magnitude and durability of these improvements.

## Introduction

Primary hyperhidrosis is a chronic disorder of sweating beyond thermoregulatory requirements that commonly affects the axillae, palms, soles, and craniofacial regions. It affects approximately 2%–5% of the population and can substantially disrupt social, occupational, and daily functioning, with reported associations with anxiety, depressive symptoms, and reduced self-esteem (1–3). In hot climates such as Saudi Arabia, environmental conditions may further intensify its burden, supporting the need for locally relevant data (4).

Conservative treatments include topical aluminum chloride, oral anticholinergics, iontophoresis, and botulinum toxin injections (5). For severe focal disease that remains inadequately controlled, sympathetic surgery can provide rapid symptom relief. International and regional studies, including a previous Saudi study of upper-limb hyperhidrosis, have reported high efficacy, improved postoperative QoL, and high satisfaction (4, 6–8). Compensatory hyperhidrosis is the most frequent adverse effect and may influence the overall assessment of benefit (8, 9).

Validated Arabic versions of the Hyperhidrosis Disease Severity Scale (Ar-HDSS) and Hyperhidrosis Quality of Life Index (HidroQoL©) enable culturally appropriate, disease-specific assessment. The Ar-HDSS provides a four-point measure of disease severity, while the HidroQoL measures daily-life and psychosocial impairment (10, 11).

Region-specific evidence using these validated Arabic patient-reported outcome measures after sympathectomy remains limited. This study therefore compared current postoperative disease severity and QoL with retrospectively recalled preoperative status among participants who reported undergoing sympathectomy. The analysis was based on a cross-sectional survey with paired within-participant comparisons; it was not a prospective pre-post cohort study.

## Materials and methods

### Study design, setting, and participants

This study was a secondary analysis of a cross-sectional survey conducted across Saudi Arabia between May 2024 and April 2025. All study variables were collected at one survey time point. The same Arabic electronic questionnaire was distributed through a Telegram support community for individuals with hyperhidrosis and through dermatology clinics at King Saud University Medical Center in Riyadh. Participation was voluntary, and respondents were identified through their questionnaire submissions. Eligibility for the present secondary analysis was assessed from self-reported questionnaire data and required reported previous sympathectomy together with complete current-postoperative and retrospectively recalled preoperative patient-reported outcome measures.

Participants in the present analysis overlap with the larger hyperhidrosis cohort reported previously (12). The previous publication examined cross-sectional disease severity, anatomical involvement, and quality-of-life burden in the complete cohort and did not evaluate paired patient-reported outcomes before and after sympathectomy. The objectives and analyses of the present study are therefore distinct.

### Sympathectomy subgroup

The present analysis included 94 respondents from the 276-person source dataset who answered “yes” to the questionnaire item asking whether they had undergone surgery for hyperhidrosis (with sympathectomy or a similar procedure given as examples). All 94 had complete responses to the recalled preoperative and current postoperative Ar-HDSS and all 18 HidroQoL© items; there were no missing item responses and no imputation. The study subgroup was reported to have undergone bilateral sympathectomy; surgical status relied on participant report. Operative records were unavailable for independent verification. Both assessments were completed at the same postoperative survey, allowing paired within-participant comparison but not a prospective pre-post evaluation. The questionnaire recorded surgery for hyperhidrosis but did not establish the site-specific operative indication, distinguish tissue excision (sympathectomy) from nerve division (sympathicotomy), or record the level of sympathetic interruption. Reported affected anatomical sites describe disease distribution and should not be interpreted as confirmed surgical indications. “Sympathectomy” is used as the reported procedure category; it does not establish endoscopic thoracic access or a particular interruption technique.

### Study questionnaire

The survey comprised three major components:

1. Sociodemographic variables: age, sex, educational level, occupation, and monthly income
2. Medical and disease-related history: comorbidities, age at hyperhidrosis onset, affected anatomical sites, and previous treatment attempts
3. Validated disease-specific arabic instruments: the ar-HDSS and hidroQoL

For the sympathectomy subgroup, both instruments were administered electronically at a single postoperative survey. Participants reported their current postoperative status and, during that same survey, retrospectively recalled their preoperative status using the same measures. The questionnaire contained no dedicated questions about compensatory sweating, recurrence, or other postoperative complications; these outcomes were not systematically assessed. It contained no dedicated questions on the operative technique or level of sympathetic interruption. The date of surgery and site-specific surgical indication were not recorded.

### Hyperhidrosis disease severity scale

The Hyperhidrosis Disease Severity Scale (HDSS), originally developed by Kowalski and colleagues in 2004, is a single-item patient-reported measure of sweating severity (13). Participants select one of four statements describing the impact of sweating on daily activities, ranging from “*never noticeable and never interfering”* to “*intolerable and always interfering.”* Scores of 3 or 4 indicate severe hyperhidrosis, whereas scores of 1 or 2 indicate mild-to-moderate disease. The validated Arabic version (Ar-HDSS) was used throughout the study (11).

### Hyperhidrosis quality of life index

The HidroQoL©, developed by Kamudoni et al. in 2015, assesses the impact of hyperhidrosis on daily functioning and psychosocial well-being. The instrument comprises 18 items across two domains and uses a three-point response scale (0 = not at all, 1 = a little, and 2 = very much). Total scores range from 0 to 36, with higher scores indicating greater impairment (14). For interpretation, total scores were grouped using the banding system of Donhauser et al., which describes the effect on the patient's life as none (0–6), small (7–18), moderate (19–25), very large (26–32), or extremely large (33–36) (15).

The validated Arabic adaptation of the HidroQoL© was used for both current postoperative status and retrospectively recalled preoperative status. The daily-life domain comprises six items (range, 0–12), and the psychosocial domain comprises twelve items (range, 0–24), including the item “My appearance is affected.” The total score is the sum of all 18 items (range, 0–36). Domain and total scores were calculated separately for each assessment using this item allocation (10, 14).

### Ethical considerations

Ethical approval was obtained from the Institutional Review Board of the College of Medicine, King Saud University, in February 2024. Electronic informed consent was obtained from all participants after providing information regarding the study purpose, estimated completion time, investigator contact details, and the voluntary nature of participation. No personally identifiable information was collected, and no incentives were offered.

### Statistical analysis

Statistical analyses were performed using IBM SPSS Statistics version 27; effect sizes and their confidence intervals were additionally computed in Python 3.12.14 with the SciPy library. Continuous variables were summarized as means and standard deviations (SD) and, for the paired outcomes, also as medians and interquartile ranges (IQR). Categorical variables were summarized as frequencies and percentages. The primary outcomes were the HidroQoL© daily-life, psychosocial, and total scores, and the Ar-HDSS grade was a secondary outcome. For each participant, the paired change was calculated as the current postoperative score minus the recalled preoperative score, so negative values indicate less impairment after surgery.

The distribution of the paired differences was examined with the Shapiro–Wilk test and histograms. Because the differences departed from normality for every outcome (all *p* < 0.001), the Wilcoxon signed-rank test was used as the primary paired test. Zero differences were excluded from ranking, and the normal approximation with correction for ties was used. Its effect size was the matched-pairs rank-biserial correlation (*r_rb_*), with a 95% CI from 10,000 percentile bootstrap resamples, and the paired location shift was estimated with the Hodges–Lehmann estimator and a signed-rank-based 95% CI. Paired t tests were reported alongside as a sensitivity analysis, together with the mean paired change, its 95% CI, and Cohen's *d_z_*, calculated as the mean of the paired differences divided by their SD; the 95% CI of *d_z_* was obtained from the noncentral t distribution. For the Ar-HDSS, the proportion of participants with severe disease (Grade 3 or 4) at recalled preoperative and current postoperative assessment was compared with the exact McNemar test.

All 94 participants had complete paired recalled preoperative and current postoperative data, so no values were missing and none were imputed. No *a priori* sample-size calculation was performed because this was a secondary analysis of a fixed dataset. All tests were two-sided, and *p* < 0.05 was considered statistically significant.

## Results

### Demographic and clinical characteristics

Table 1 summarizes the demographic and clinical characteristics of the 94 participants who reported undergoing sympathectomy. The mean age at survey completion was 30.7 ± 8.9 years, with a median age of 29 years (range, 16–63 years). Most participants were aged 25–39 years (54.2%). Age distribution was as follows: <20 years, 6 (6.4%); 20–24 years, 20 (21.3%); 25–29 years, 24 (25.5%); 30–39 years, 27 (28.7%); and ≥40 years, 17 (18.1%). The date of surgery was not recorded, so the interval between surgery and survey completion could not be calculated.

**Table 1:** Demographic and clinical characteristics of the sympathectomy cohort (*N* = 94).

Characteristic	*n*	%
Age, years		
<20	6	6.4
20–24	20	21.3
25–29	24	25.5
30–39	27	28.7
40+	17	18.1
Mean ± SD	30.7 ± 8.9	
Sex		
Male	60	63.8
Female	34	36.2
Nationality		
Saudi	84	89.4
Non-Saudi	10	10.6
Educational level		
Secondary school or below	16	17.0
University graduate	68	72.3
Postgraduate	10	10.6
Occupation		
Unemployed	17	18.1
Student	13	13.8
Non-healthcare field	58	61.7
Healthcare field	6	6.4
Monthly income, SAR		
<5,000	35	37.2
5,000–15,000	34	36.2
15,000–30,000	21	22.3
>30,000	4	4.3
Comorbidities		
None	85	90.4
Diabetes mellitus	3	3.2
Hypertension	0	0.0
Hypercholesterolemia	1	1.1
Hypothyroidism	2	2.1
Other	2	2.1
Age at onset of hyperhidrosis		
Since birth	22	23.4
1–9 years	19	20.2
10–15 years	28	29.8
16–20 years	16	17.0
>20 years	9	9.6
Mean ± SD, years	11.2 ± 9.2	

SD, standard deviation; SAR, Saudi Arabian Riyal.

Men comprised 60 participants (63.8%) and women 34 (36.2%), and most participants were Saudi nationals (84, 89.4%). Most participants had a university-level education (72.3%), whereas 16 (17.0%) had secondary education or lower and 10 (10.6%) had postgraduate education.

Regarding employment status, 58 participants (61.7%) were employed in non-healthcare occupations, 6 (6.4%) worked in healthcare, 13 (13.8%) were students, and 17 (18.1%) were unemployed. Monthly income was distributed as follows: <5,000 Saudi Arabian Riyal (SAR), 35 (37.2%); 5,000–15,000 SAR, 34 (36.2%); 15,000–30,000 SAR, 21 (22.3%); and >30,000 SAR, 4 (4.3%). Comorbidities were uncommon, with 85 patients (90.4%) reporting no underlying medical conditions.

The mean age at symptom onset was 11.2 ± 9.2 years. The most frequently reported age at onset was 10–15 years (28, 29.8%), followed by birth (22, 23.4%), 1–9 years (19, 20.2%), 16–20 years (16, 17.0%), and >20 years (9, 9.6%).

### Affected areas and previous management

Across the sympathectomy cohort, participants reported hyperhidrosis affecting the palms, soles, axillae, face/scalp, trunk, and genital region. Previous management methods are also summarized in Figure 1.

**Figure 1:**
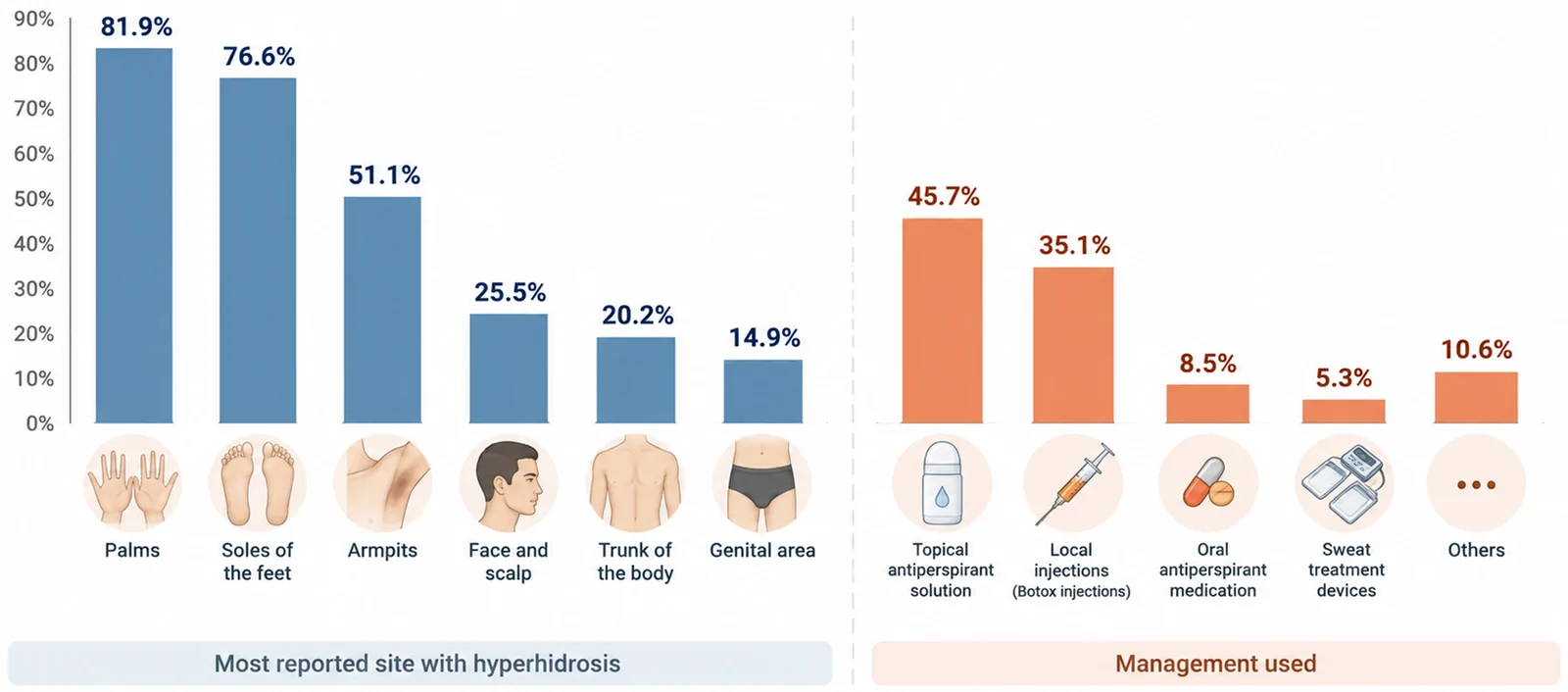
Affected sites and previous treatments in the sympathectomy cohort. Percentages are based on 94 participants; participants could report multiple sites and treatments.

### Disease severity

Recalled preoperative disease severity was predominantly high (Figure 2), with 13 participants (13.8%) classified as Grade 2, 26 (27.7%) as Grade 3, and 55 (58.5%) as Grade 4; no participants were classified as Grade 1. At the current postoperative assessment, 35 participants (37.2%) were classified as Grade 1, 22 (23.4%) as Grade 2, 18 (19.1%) as Grade 3, and 19 (20.2%) as Grade 4 (Figure 3). The median Ar-HDSS grade fell from 4 (IQR 3–4) to 2 (IQR 1–3) (Wilcoxon signed-rank test, z = −6.43, *p* < 0.001; *r_rb_* = −0.98, 95% CI −1.00 to −0.94). Fifty-three participants (56.4%) improved by at least one grade, 39 (41.5%) reported the same grade, and 2 (2.1%) reported a worse grade. The proportion with severe disease (Grade 3 or 4) decreased from 81 (86.2%) to 37 (39.4%) (exact McNemar test, *p* < 0.001). Of the 55 participants with recalled Grade 4 disease, 18 (32.7%) still reported current postoperative Grade 4.

**Figure 2:**
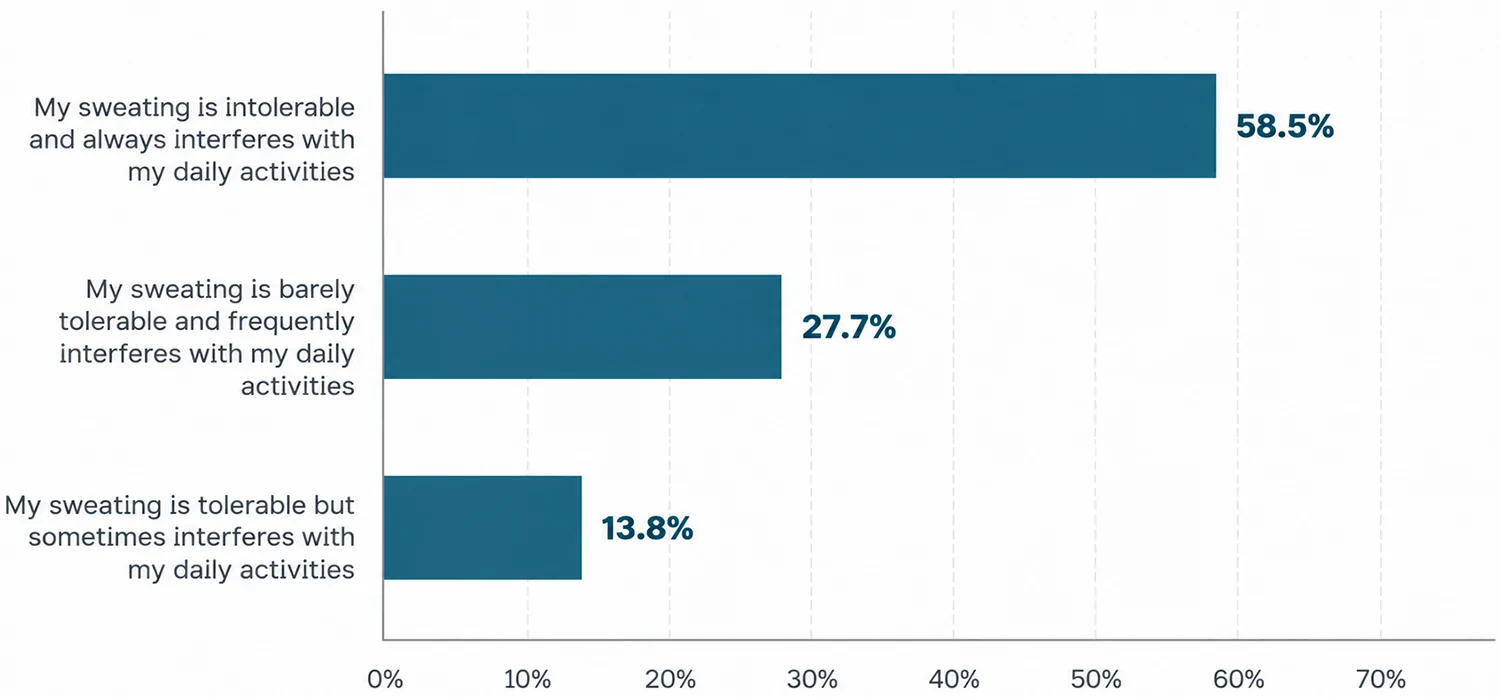
Recalled preoperative hyperhidrosis severity in the sympathectomy cohort. Percentages are based on 94 participants.

**Figure 3:**
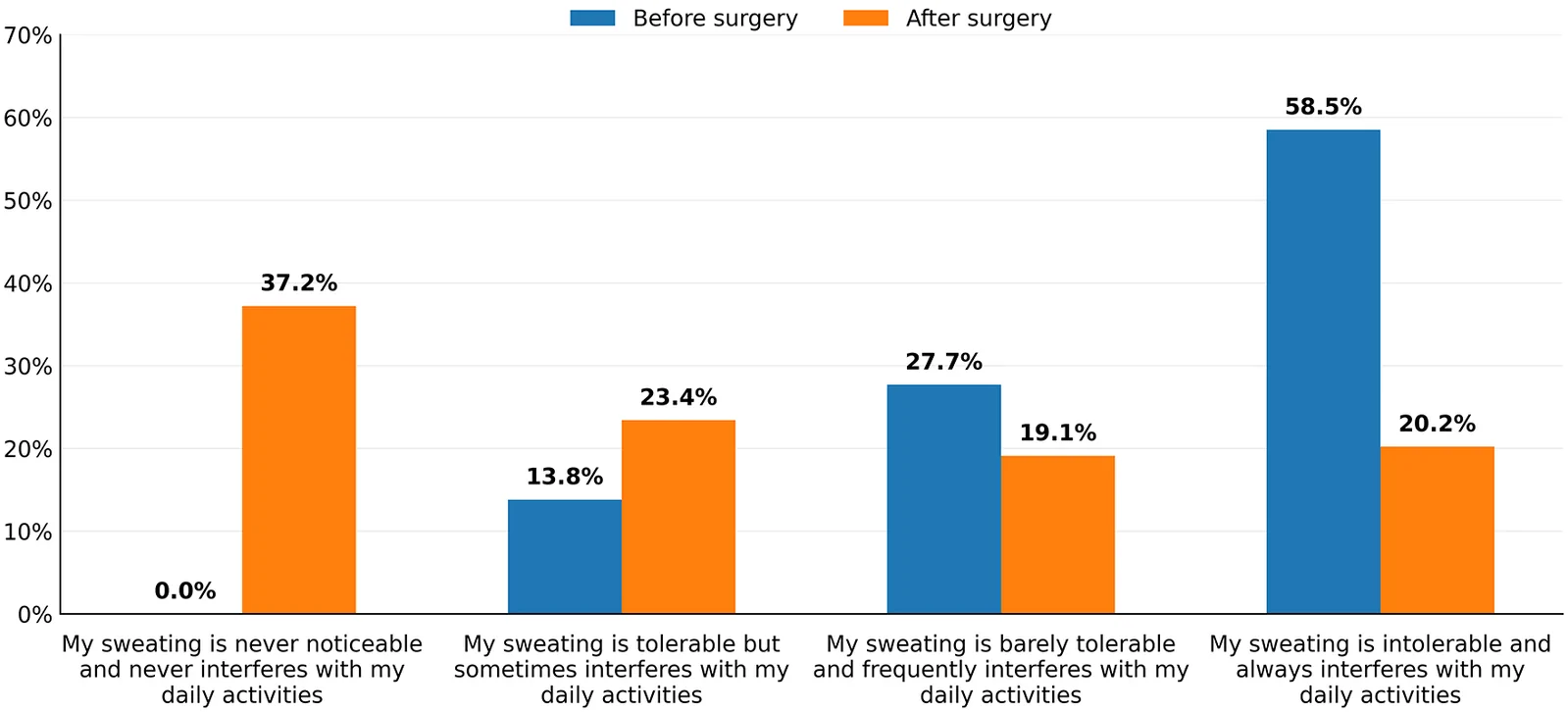
Recalled preoperative and current postoperative hyperhidrosis severity. In the figure key, “Before surgery” denotes retrospectively recalled preoperative ratings and “After surgery” denotes current postoperative ratings. Percentages are based on 94 participants.

### Quality-of-life impact

Table 2 summarizes recalled preoperative item-level burden and domain scores. The mean daily-life domain score was 8.5 ± 2.8 (scale, 0–12), and the mean psychosocial domain score was 16.9 ± 5.5 (scale, 0–24), indicating considerable functional and psychosocial burden.

**Table 2:** Recalled preoperative hyperhidrosis quality-of-life responses (*N* = 94).

Item	Not at all, *n* (%)	A little, *n* (%)	Very much, *n* (%)
Daily-life activities			
My clothing choices are affected	14 (14.9)	21 (22.3)	59 (62.8)
My physical activity is affected	24 (25.5)	27 (28.7)	43 (45.7)
My hobbies are affected	16 (17.0)	31 (33.0)	47 (50.0)
My work is affected	6 (6.4)	31 (33.0)	57 (60.6)
I worry about dealing with hyperhidrosis when doing extra activities	0 (0.0)	15 (16.0)	79 (84.0)
My holidays are affected (in terms of planning, activities, etc.)	23 (24.5)	37 (39.4)	34 (36.2)
Daily-life domain score, min–max (mean ± SD)	2–12 (8.5 ± 2.8)		
Psychosocial life			
I feel stressed	2 (2.1)	25 (26.6)	67 (71.3)
I feel embarrassed	2 (2.1)	17 (18.1)	75 (79.8)
I feel frustrated	14 (14.9)	24 (25.5)	56 (59.6)
I am bothered by physically expressing emotions (like hugging)	17 (18.1)	19 (20.2)	58 (61.7)
Hyperhidrosis is constantly on my mind	4 (4.3)	18 (19.1)	72 (76.6)
I worry about my future health	28 (29.8)	31 (33.0)	35 (37.2)
I worry about how others will react	5 (5.3)	18 (19.1)	71 (75.5)
I worry about leaving sweat stains on things	2 (2.1)	14 (14.9)	78 (83.0)
I avoid meeting new people	25 (26.6)	30 (31.9)	39 (41.5)
I avoid public speaking (like giving presentations)	20 (21.3)	30 (31.9)	44 (46.8)
My appearance is affected	23 (24.5)	30 (31.9)	41 (43.6)
My sexual life is affected	55 (58.5)	20 (21.3)	19 (20.2)
Psychosocial domain score, min–max (mean ± SD)	1–24 (16.9 ± 5.5)		

SD, standard deviation.

Table 3 presents the paired comparisons for all 94 participants. The daily-life domain score decreased from 8.5 ± 2.8 (median 9.0, IQR 6.0–11.0) for recalled preoperative status to 4.9 ± 4.5 (median 3.5, IQR 0.3–10.0) for current postoperative status. The mean paired change was −3.6 points (95% CI −4.5 to −2.8), and the Hodges–Lehmann estimate was −3.5 (95% CI −4.5 to −2.5) (Wilcoxon signed-rank test, z = −6.42, *p* < 0.001; *r_rb_* = −0.88, 95% CI −0.96 to −0.76). The psychosocial domain score decreased from 16.9 ± 5.5 (median 17.5, IQR 13.3–21.8) to 8.8 ± 8.7 (median 5.5, IQR 0.3–16.0), with a mean paired change of −8.1 points (95% CI −9.7 to −6.4) and a Hodges–Lehmann estimate of −8.0 (95% CI −9.5 to −6.5) (z = −6.84, *p* < 0.001; *r_rb_* = −0.88, 95% CI −0.95 to −0.77). The total HidroQoL© score decreased from 25.4 ± 7.5 (median 27.0, IQR 20.0–31.0) to 13.7 ± 12.8 (median 11.0, IQR 1.3–25.8), with a mean paired change of −11.7 points (95% CI −14.1 to −9.3) and a Hodges–Lehmann estimate of −12.0 (95% CI −14.0 to −9.5) (z = −6.90, *p* < 0.001; *r_rb_* = −0.88, 95% CI −0.95 to −0.78). Paired t tests led to the same conclusions, with *d_z_* values of −0.88 (95% CI −1.11 to −0.64), −0.97 (95% CI −1.22 to −0.73), and −0.98 (95% CI −1.23 to −0.74) for the daily-life, psychosocial, and total scores, respectively.

**Table 3:** Recalled preoperative and current postoperative hidroQoL© and ar-HDSS scores with paired comparisons (*N* = 94 complete pairs).

Outcome (score range)	Recalled preoperative value	Current postoperative value	Paired change (95% CI)	Effect size (95% CI)	Statistical test	*p* value
HidroQoL© daily-life (0–12) Median (IQR)	9.0 (6.0–11.0)	3.5 (0.3–10.0)	HL −3.5 (−4.5 to −2.5)	*r_rb_* −0.88 (−0.96 to −0.76)	Wilcoxon z = −6.42	<0.001
Mean ± SD	8.5 ± 2.8	4.9 ± 4.5	MD −3.6 (−4.5 to −2.8)	*d_z_* −0.88 (−1.11 to −0.64)	Paired t t(93) = −8.49	<0.001
HidroQoL© psychosocial (0–24) Median (IQR)	17.5 (13.3–21.8)	5.5 (0.3–16.0)	HL −8.0 (−9.5 to −6.5)	*r_rb_* −0.88 (−0.95 to −0.77)	Wilcoxon z = −6.84	<0.001
Mean ± SD	16.9 ± 5.5	8.8 ± 8.7	MD −8.1 (−9.7 to −6.4)	*d_z_* −0.97 (−1.22 to −0.73)	Paired t t(93) = −9.44	<0.001
HidroQoL© total (0–36) Median (IQR)	27.0 (20.0–31.0)	11.0 (1.3–25.8)	HL −12.0 (−14.0 to −9.5)	*r_rb_* −0.88 (−0.95 to −0.78)	Wilcoxon z = −6.90	<0.001
Mean ± SD	25.4 ± 7.5	13.7 ± 12.8	MD −11.7 (−14.1 to −9.3)	*d_z_* −0.98 (−1.23 to −0.74)	Paired t t(93) = −9.54	<0.001
Ar-HDSS grade (1–4) Median (IQR)	4 (3–4)	2 (1–3)	Improved 53 (56.4%); unchanged 39 (41.5%); worse 2 (2.1%)	*r_rb_* −0.98 (−1.00 to −0.94)	Wilcoxon z = −6.43	<0.001

Both ratings were obtained at the same postoperative survey; all 94 participants had complete paired data. Change is current postoperative minus recalled preoperative score. Wilcoxon signed-rank tests were primary; zero differences were excluded (nonzero pairs: daily-life 71, psychosocial 80, total 81, Ar-HDSS 55), with average ranks for ties and tie-adjusted normal approximations without continuity correction. Paired t tests (df = 93) were sensitivity analyses. Mean-change CIs use the t distribution; SDs of paired differences were 4.2, 8.3, and 11.9 points for daily-life, psychosocial, and total scores. Cohen's *d_z_* CIs use the noncentral t distribution; *r_rb_* CIs use 10,000 participant-level percentile bootstrap resamples. HL is the median Walsh-average paired location shift, with signed-rank-based CIs; ties and asymmetry may affect interpretation and interval coverage. Negative effects indicate lower current postoperative scores. All *p* values are two-sided and unadjusted. CI, confidence interval; *d_z_*, Cohen's paired standardized mean difference; HL, Hodges–Lehmann estimate; IQR, interquartile range; MD, mean paired difference; *r_rb_*, matched-pairs rank-biserial correlation; SD, standard deviation.

The response was not uniform across participants. For the total score, 68 participants (72.3%) reported lower scores after surgery, 13 (13.8%) reported no change, and 13 (13.8%) reported higher scores; 16 (17.0%) reported a postoperative total score of 0. The corresponding figures were 61 (64.9%), 23 (24.5%), and 10 (10.6%) for the daily-life domain and 69 (73.4%), 14 (14.9%), and 11 (11.7%) for the psychosocial domain. Using the HidroQoL© score bands, 51 participants (54.3%) recalled a very large or extremely large effect on their lives before surgery, compared with 24 (25.5%) at the current postoperative assessment, while the proportion in the no-effect band rose from 3 (3.2%) to 39 (41.5%).

## Discussion

In this cross-sectional survey with retrospective recall of preoperative status, participants reported lower hyperhidrosis-specific QoL impairment at the current postoperative assessment than they recalled before surgery. Daily-life scores were 8.5 ± 2.8 for recalled preoperative status and 4.9 ± 4.5 postoperatively, while psychosocial scores were 16.9 ± 5.5 and 8.8 ± 8.7, respectively. Total HidroQoL scores were 25.4 ± 7.5 and 13.7 ± 12.8, with a mean paired change of −11.7 points (95% CI, −14.1 to −9.3). Paired effect sizes were large (*d_z_* −0.88 to −0.98; *r_rb_* −0.88 across the three HidroQoL© outcomes). These findings are broadly consistent with previous Saudi and international reports of improved QoL following sympathectomy (4, 6, 7). However, the preoperative ratings were provided only after surgery and at the same time as the postoperative ratings. The observed differences therefore represent retrospective patient-reported associations and may be influenced by recall bias; they should not be interpreted as prospectively measured or causal treatment effects.

Compensatory sweating is an important postoperative outcome after sympathectomy and is frequently reported in previous studies (4, 8). Prior Saudi data suggested that compensatory hyperhidrosis did not necessarily reduce postoperative satisfaction or QoL (4). In the present survey, however, compensatory sweating was not systematically assessed, and no standardized information was collected regarding its presence, severity, anatomical distribution, or effect on QoL. Recurrence and other postoperative complications were likewise not systematically assessed. In addition, operative reports were unavailable and the exact surgical technique and level of sympathetic interruption could not be verified. Published surgical series illustrate the importance of documenting operative characteristics and postoperative outcomes (16–18). Therefore, these data cannot estimate postoperative complication rates or support procedure-specific conclusions about the balance between QoL outcomes and adverse effects.

The group-level improvement should be read alongside the variation between participants. About one in seven participants reported a higher total HidroQoL© score after surgery, and 37 (39.4%) still described their sweating as barely tolerable or intolerable on the Ar-HDSS. Because the HDSS asks about sweating in general rather than at the treated site, persistent high grades may partly reflect compensatory sweating rather than failure at the target site, a pattern described in earlier surgical series (8, 9). Differences in the procedure performed, its extent, and the time elapsed since surgery may also contribute. These factors were not systematically recorded in the present dataset, so this explanation cannot be tested here, but the finding is relevant when counselling patients about the benefit they can reasonably expect.

These findings support the use of validated Arabic patient-reported outcome measures when assessing patients after sympathectomy. Future prospective studies should obtain standardized measurements before surgery and document the surgical indication, operative technique, level of sympathetic interruption, compensatory sweating, recurrence, other complications, and longer-term outcomes.

A major strength of this study is the use of validated Arabic versions of the HDSS and HidroQoL, which enabled culturally and linguistically appropriate assessment of disease severity and QoL. Additional strengths include within-participant comparison of current postoperative status with recalled preoperative status and separate evaluation of daily-life and psychosocial burden. These features provide a patient-centered description of reported postoperative outcomes while not replacing prospectively collected baseline measurements.

This study has several limitations. First, preoperative HDSS and HidroQoL ratings were reconstructed retrospectively at the same postoperative survey in which current outcomes were reported. Participants' present symptoms, satisfaction, expectations, and time since surgery may therefore have influenced their recollection of preoperative burden. The magnitude and even the direction of the observed within-participant differences may be affected by recall bias; consequently, the comparisons cannot be interpreted as prospectively measured treatment effects. Using validated Arabic instruments does not remove this recall-related limitation. Second, recruitment through a voluntary online support community and university dermatology clinics used non-probability sampling, and eligibility was based on self-reported questionnaire responses, creating potential selection and misclassification bias. The available survey export does not document independent clinical confirmation of primary hyperhidrosis or exclusion of secondary causes. Third, the absence of a control group and prospectively collected baseline data further limits causal inference. Fourth, the cross-sectional survey provides only one postoperative assessment. The date of surgery was not recorded, so the length of postoperative follow-up is unknown and the durability of the reported differences cannot be judged. Fifth, operative reports were unavailable, and the surgical indication, exact technique (sympathectomy versus sympathicotomy), and level of sympathetic interruption could not be verified. These factors may affect both efficacy and the risk of compensatory sweating. Sixth, compensatory sweating, recurrence, and other postoperative complications were not systematically assessed, preventing a balanced evaluation of benefits and harms. Finally, the modest sample size and self-reported nature of the data may limit generalizability.

## Conclusion

Participants reported lower current postoperative daily-life and psychosocial impairment than they retrospectively recalled before surgery. These cross-sectional, recall-based comparisons support a possible postoperative benefit but do not establish a causal treatment effect. Prospective studies with true preoperative baseline measurements, documented operative technique and interruption level, systematic assessment of compensatory sweating, recurrence, and other complications, and longer follow-up are required to confirm the magnitude and durability of these findings.

## Data Availability

The original contributions presented in the study are included in the article/Supplementary Material, further inquiries can be directed to the corresponding author.
